# Automating creativity assessment with *SemDis*: An open platform for computing semantic distance

**DOI:** 10.3758/s13428-020-01453-w

**Published:** 2020-08-31

**Authors:** Roger E. Beaty, Dan R. Johnson

**Affiliations:** 1 Department of Psychology, Pennsylvania State University, 140 Moore Building, University Park, PA 16802 USA; 2 Department of Cognitive and Behavioral Science, Washington and Lee University, Lexington, VA 24450 USA

**Keywords:** Assessment, Creativity, Divergent thinking, Semantic distance, Word association

## Abstract

Creativity research requires assessing the quality of ideas and products. In practice, conducting creativity research often involves asking several human raters to judge participants’ responses to creativity tasks, such as judging the novelty of ideas from the alternate uses task (AUT). Although such subjective scoring methods have proved useful, they have two inherent limitations—labor cost (raters typically code thousands of responses) and subjectivity (raters vary on their perceptions and preferences)—raising classic psychometric threats to reliability and validity. We sought to address the limitations of subjective scoring by capitalizing on recent developments in automated scoring of verbal creativity via semantic distance, a computational method that uses natural language processing to quantify the semantic relatedness of texts. In five studies, we compare the top performing semantic models (e.g., GloVe, continuous bag of words) previously shown to have the highest correspondence to human relatedness judgements. We assessed these semantic models in relation to human creativity ratings from a canonical verbal creativity task (AUT; Studies 1–3) and novelty/creativity ratings from two word association tasks (Studies 4–5). We find that a latent semantic distance factor—comprised of the common variance from five semantic models—reliably and strongly predicts human creativity and novelty ratings across a range of creativity tasks. We also replicate an established experimental effect in the creativity literature (i.e., the serial order effect) and show that semantic distance correlates with other creativity measures, demonstrating convergent validity. We provide an open platform to efficiently compute semantic distance, including tutorials and documentation (https://osf.io/gz4fc/).

**Electronic supplementary material:**

The online version of this article (10.3758/s13428-020-01453-w) contains supplementary material, which is available to authorized users.

Creativity researchers have long grappled with how to measure creativity. Indeed, the question of how to best capture creativity remains open and active, with a recent special issue on creativity assessment recently published in *Psychology of Aesthetics, Creativity, and the Arts* (Barbot, Hass, & Reiter-Palmon, 2019). Over the years, a range of assessment approaches have been developed, from methods that rely on experts to judge the creative quality of products (i.e., the Consensual Assessment Technique; Amabile, 1983; Cseh & Jeffries, 2019) to frequency-based methods that use standardized norms (Forthmann, Paek, Dumas, Barbot, & Holling, 2019; Torrance, 1972) to subjective scoring methods that rely on layperson judgements (Silvia et al., 2008). Although each method has shown some degree of utility for creativity research, each comes with challenges and limitations. Two challenges that are common to most creativity assessments are *subjectivity* (raters don’t always agree on what’s creative) and *labor cost* (raters often have to score thousands of responses by hand)—both of which pose threats to the reliable and valid assessment of creativity (Barbot, 2018; Forthmann et al., 2017; Reiter-Palmon, Forthmann, & Barbot, 2019). To address these issues, researchers have begun to explore whether the process of scoring responses for their creative quality can be automated and standardized using computational methods, and preliminary evidence suggests that such tools can yield reliable and valid indices of creativity (Acar & Runco, 2014; Dumas, Organisciak, & Doherty, 2020; Heinen & Johnson, 2018; Kenett, 2019; Prabhakaran, Green, & Gray, 2014). In the present research, we aim to capitalize on these promising advances by developing and validating an open-source platform for the automated assessment of creativity, allowing researchers to objectively quantify the creative quality of ideas across a range of common creativity tasks.

## Measuring creativity: The status quo

Creative thinking is widely assessed with tests of divergent thinking, which present open-ended prompts and ask people to think of creative responses (Acar & Runco, 2019). One of the most widely used tests of divergent thinking is the Alternate Uses Task (AUT), where people are presented a common object (e.g., a box) and asked to think of as many creative and uncommon uses for it as possible within a given time period (usually 2–3 min; Benedek, Mühlmann, Jauk, & Neubauer, 2013). A strength of the AUT is that it seems to offer a good approximation of a person’s general capacity to come up with original ideas. Although it has limitations (Barbot, 2018), the AUT and other divergent thinking tests have shown consistent evidence of validity, with several studies reporting moderate to large correlations between AUT performance and real-world creative achievement in the arts and sciences (Beaty et al., 2018; Jauk, Benedek, & Neubauer, 2014; Plucker, 1999). Paul Torrance, who developed a widely used creativity assessment (the Torrance Test of Creative Thinking; TTCT), provided perhaps the most compelling longitudinal evidence for the validity of divergent thinking tests: highly creative children—assessed by performance on the TTCT—grew up to be highly creative adults, reporting significantly more creative accomplishments when assessed decades later in adulthood (Plucker, 1999; Torrance, 1981); remarkably, a 50-year follow-up of Torrance’s data further confirmed the validity of divergent thinking in predicting students’ future creative accomplishment (Runco, Millar, Acar, & Cramond, 2010). These findings indicate that divergent thinking tests provide a measure of “domain-general” creative ability that may support real-world creative actions (cf. Jauk et al., 2014; but see Barbot, Besançon, & Lubart, 2016; Dietrich, 2015; and Zeng, Proctor, & Salvendy, 2011 for alternate views on the domain-generality and utility of divergent thinking tasks).

Divergent thinking responses are often scored on two dimensions: fluency (the total number of responses) and originality (the creative quality of responses). Fluency offers a proxy of generative ability; however, it has been criticized for a lack of reliability, with inter-item fluency correlations on the AUT often as low as .3 to .4 (Barbot, 2018; cf. Dumas & Dunbar, 2014). Recent work suggests that this low inter-item correlation could be due to variability in item (object) characteristics such as semantic object features (Beaty, Kenett, Hass, & Schacter, 2019) and word frequency (Forthmann et al., 2016). At the same time, low inter-task fluency correlations have not been consistently reported in the literature; for example, Jauk et al. (2014) reported high standardized factor loadings on an AUT fluency latent variable (suggesting strong reliability) and Forthmann, Holling, Çelik, Storme, and Lubart (2017) reported inter-task correlations for AUT items ranging from .57 to .71. Nevertheless, perhaps the most notable limitation of fluency is that it does not take into consideration the quality of ideas. Thus, a given person may produce many ideas on the AUT—which would be captured by calculating their fluency score—but, absence an index of quality, whether those ideas were actually creative (i.e., qualitatively different from common ideas) would be unknown.

Originality scoring, in contrast, can capture the creative quality of responses. A popular approach to originality scoring is the subjective scoring method (Hass, Rivera, & Silvia, 2018; Silvia et al., 2008). The subjective scoring method is based on the Consensual Assessment Technique (CAT; Amabile, 1983; Cseh & Jeffries, 2019; Kaufman, Lee, Baer, & Lee, 2007), a procedure that involves convening a panel of experts to judge a series of products, ranging from ideas to poems to inventions. When applied to divergent thinking assessment via the subjective scoring method, a group of raters (often undergraduate students) are briefly trained on how to assess the creative quality of responses, typically using a 1 (*not at all creative*) to 5 (*very creative*) scale (Benedek et al., 2013; Silvia et al., 2008). Notably, the subjective scoring method, like the CAT, provides only limited guidance to raters as to what constitutes a creative response (e.g., uncommon, remote, clever), largely deferring to raters’ own subjective perception of creativity (Cseh & Jeffries, 2019; Mouchiroud & Lubart, 2001). Although subjective scoring methods have shown evidence of convergent validity, including positive correlations with frequency-based originality (Forthmann, Holling, Çelik, et al., 2017) and measures of creative activities and achievements (Jauk et al., 2014), inter-rater agreement is not always high, raising issues of reliability (Barbot, 2018). Reconciling such disagreements is a common feature of the CAT—where experts can meet to discuss their ratings and work toward agreement—but many studies using subjective scoring with divergent thinking responses do not employ this approach, likely due to its time-consuming nature. Moreover, the undergraduate students that often serve as raters for these tests are typically tasked with scoring thousands of responses, leading to rater fatigue and contributing to poor reliability (Forthmann, Holling, Zandi, et al., 2017). Taken together, although the CAT and subjective scoring method have been valuable to creativity research, the approaches are marked by the key limitations of subjectivity and labor cost.

## Automating creativity assessment

To address the limitations of subjective scoring, researchers have begun to explore the utility of automated scoring approaches using computational tools (Acar & Runco, 2014; Dumas et al., 2020; Dumas & Runco, 2018; Green, 2016; Hass, 2017b; Heinen & Johnson, 2018; Kenett, 2019; Prabhakaran et al., 2014; Zedelius, Mills, & Schooler, 2019). One such approach uses latent semantic analysis (LSA; Landauer, Foltz, & Laham, 1998) to quantify the “semantic distance” between concepts in a given semantic space. LSA and other computational linguistic tools can quantify the semantic relatedness between words in large corpora of texts, for example, by counting the number of co-occurrences between words and documents (i.e., count models) or by deriving co-occurrence weights by trying to predict word-context links (i.e., predict models), all in a high-dimensional word-vector space (Günther, Rinaldi, & Marelli, 2019). For example, the words “hammer” and “nail” are likely to occur in similar contexts and would thus yield a higher similarity value; in contrast, the words “hammer” and “tissue” are less likely to occur in similar contexts and would thus yield a relatively lower similarity value. Application of LSA in creativity research is rooted in the associative theory of creativity (Kenett, 2019; Mednick, 1962) which proposes that creative thinking requires making connections between seemingly “remote” concepts. The associative theory has received increasing support from several recent computational modeling studies showing that high-creative individuals, defined by performance on a battery of creativity tasks, show a more flexible semantic network structure, characterized by low modularity and high connectivity between concepts (Christensen, Kenett, Cotter, Beaty, & Silvia, 2018; Gray et al., 2019; Kenett et al., 2018; Kenett, Anaki, & Faust, 2014; Kenett & Faust, 2019). According to Kenett and colleagues, this flexible (or small-world) semantic network architecture is conducive to creative thinking because it allows people to form conceptual combinations between concepts that are typically represented further apart (e.g., hammer and tissue).

Prabhakaran et al. (2014) provided an early test of LSA for creativity assessment in the context of the classic verb generation task (see also Bossomaier, Harre, Knittel, & Snyder, 2009; Forster & Dunbar, 2009). When presented with nouns and instructed to “think creatively” while searching for verbs to relate to the nouns, participants produced responses that were significantly more semantically distant, defined as the inverse of semantic similarity, compared to when they were not cued to think creatively (and simply generated common verbs). Here, the simple instruction to “think creatively” yielded more creative (i.e., semantically distant) responses, consistent with prior work showing explicit instruction to think creatively improves creative task performance (Acar, Runco, & Park, 2019; Nusbaum, Silvia, & Beaty, 2014; Said-Metwaly, Fernández-Castilla, Kyndt, & Van den Noortgate, 2019). Critically, at the individual subject level, the authors found that semantic distance values in the cued creativity condition correlated positively with a range of established creativity measures, including human ratings of creativity on divergent thinking tests, performance on a creative writing task, and frequency of self-reported creative achievement in the arts and sciences. Prabhakaran et al. (2014) thus provided validity evidence of LSA for creativity research in the context of the verb generation task, demonstrating the potential of using automated scoring approaches to measure verbal creativity.

The initial LSA findings of Prabhakaran et al. (2014) have since been replicated using a different computational model and corpora (Heinen & Johnson, 2018) and extended to other creativity tasks, including the AUT (Hass, 2017b), albeit with mixed evidence for validity (Forster & Dunbar, 2009; Forthmann, Holling, Çelik, et al., 2017; Forthmann, Oyebade, Ojo, Günther, & Holling, 2018; Harbison & Haarmann, 2014; Hass, 2017a; Hass, 2017b). As LSA has been increasingly employed in creativity research, researchers have begun to identify limitations of the approach and best-practices in data processing. In a study on the AUT, for example, Forthmann et al. (2019) found that LSA values are confounded by elaboration—the more words used to describe a response, the higher LSA-based cosine similarity (i.e., lower semantic distance derived from similarity)—but this confound was partially mitigated by removing “stop words” from responses (e.g., the, an, but) prior to computing LSA. Another consideration with semantic distance-based scoring concerns the balance of novelty and usefulness (or appropriateness), the two criteria that jointly define a creative idea or product (Diedrich, Benedek, Jauk, & Neubauer, 2015). In addition to detecting novelty, Heinen and Johnson (2018) found that LSA can also be used to assess the combination of novelty and usefulness/appropriateness, depending on the type of instruction given to participants: semantic distance was lowest with a “common” instruction, highest with a “random” instruction, and between common and random with a “creative” instruction. They found that when participants were asked to “be creative,” they spontaneously tended to give creative responses constrained by appropriateness, as opposed to giving highly novel, but nonsensical responses. These findings demonstrate the utility of semantic distance metrics as a means to quantify creativity in the context of verbal idea generation tasks.

## The present research

Subjective scoring methods are commonly used to assess the creative quality of responses on verbal creativity tasks. Although subjective methods and other manual-based approaches have shown evidence of reliability and validity (Silvia et al., 2008), they suffer from two fundamental issues: subjectivity and labor cost. Regarding subjectivity, raters don’t always agree on what constitutes a creative response, and they are often given little guidance—consistent with the widely adopted guidelines of the Consensual Assessment Technique (Cseh & Jeffries, 2019)—leading to low inter-rater reliability. Moreover, raters are often asked to code hundreds or thousands of responses, leading to rater fatigue and further threatening reliability (Forthmann, Holling, Zandi, et al., 2017). Critically, these issues can also act as a barrier of entry for people without the time and resources to code thousands of responses by hand, such as researchers without teams of research assistants, or educators without the time to score creativity tests. To address the limitations of subjective scoring methods, automated scoring methods such as LSA have begun to be employed, with preliminary evidence pointing to their potential to provide a reliable and valid index of creative thinking ability, particularly with tasks that require single word responses (Heinen & Johnson, 2018; Kenett, 2019; Prabhakaran et al., 2014), with more mixed findings for tasks that require multi-word responses, like the AUT.

In the present research, we aim to capitalize on recent progress in the automated scoring of verbal creativity. We develop and test a new online platform that computes semantic distance called *SemDis*. *SemDis* was built to handle a range of verbal creativity and association tasks, including single word associations and word phrase associations, with a focus on the AUT. SemDis compliments and extends recent efforts to compare the relative performance of various computational approaches to computing semantic distance in predicting human creativity ratings. For example, Dumas et al. compared several semantic models (TASA-LSA, EN_100k_lsa, GloVe 840B, and word2vec-skipgram) in predicting human creativity ratings on the AUT, reporting evidence for the reliability and validity of these different models, particularly GloVe (Dumas et al., 2020). Here we build on the work of Dumas and colleagues by: 1) comparing additive and multiplicative composition of vectors, 2) modeling various semantic spaces within a latent variable approach (reducing biases of any single text corpus; Kenett, 2019), 3) including multiple published and unpublished datasets, 4) considering both AUT and word association responses, and 5) including a variety of external validity criteria.

Using latent variable modeling, we extract common measurement variance across multiple metrics of semantic distance and test how well this latent factor predicts human creativity ratings. As a further test of validity, we examine whether the semantic distance factor predicts established creativity measures, including real-world creative achievement and creative self-efficacy, as well as other cognitive assessments of verbal creativity (e.g., creative metaphor production). Our goal is to provide a reliable, valid, and automated assessment of creativity. To our knowledge, we provide the first comparison between the application of additive and multiplicative compositional semantic models in the context of creativity assessment. Compositional semantic models are relevant when participants give multi-word responses (e.g., AUT) and a researcher needs to combine each individual word vector into a single compositional vector. There is some preliminary evidence that multiplicative models may show higher correlations with human ratings because, compared to an additive model, similar meanings between two responses get more weight, and dissimilar meanings get less weight in the final compositional vector (Mitchell & Lapata, 2010). In addition, prior research suggests one substantial weakness of applying an additive compositional model in creativity assessment is that it penalizes (i.e., reduces) semantic distance scores for more elaborate creativity responses (Forthmann et al., 2018). We attempt to replicate this finding and determine whether or not multiplicative models similarly penalize semantic distance scores, with the goal of explaining maximal variance in human creativity ratings.

Although similar tools are currently available (e.g., lsacolorado.edu; snaut, Mandera, Keuleers, & Brysbaert, 2017), we provide more robust text processing via optional methods of text cleaning, more flexibility in the creation of underlying semantic model (i.e., allowing users to select which semantic space and which compositional model to include in the computation of semantic distance), and latent variable-extracted factor scores from diverse semantic spaces. In addition, in contrast to some platforms, our online platform (*SemDis*) can run on Macs or PCs because it is a web-based platform and does not require downloading software.

## Study 1

Our first study aimed to provide preliminary evidence for the reliability and validity of our approach to automated creativity assessment using latent variable modeling. To this end, we test whether combining multiple models of semantic distance into a single latent variable can approximate human creativity ratings. Latent variables can suppress methodological variance specific to each model, mitigating unreliability by reducing the influence of any one semantic model and extracting common measurement variance across multiple models (cf. Beketayev & Runco, 2016). We reanalyzed AUT responses from a recently published study (Beaty et al., 2018) and tested the relative performance of five semantic models in predicting human creativity ratings. We focused on additive and multiplicative semantic models that have previously shown adequate correspondence to human ratings and semantic similarity (Mitchell & Lapata, 2010). Regarding validity, we examined the extent to which a latent variable, comprised of common variance of the five semantic models, relates to several other measures of creativity, assessed via task performance and self-report. Previous research using word association tasks and semantic distance values found that semantic distance on these tasks correlated with both human ratings (Heinen & Johnson, 2018; Johnson, Cuthbert, & Tynan, 2019) and a range of other creativity measures (Prabhakaran et al., 2014). We thus expected our combined semantic distance latent variable to positively correlate with human creativity ratings on the AUT and other creativity measures.

## Method

### Participants

Participants were recruited as part of a larger project on individual differences in creativity (see Adnan, Beaty, Silvia, Spreng, & Turner, 2019; Beaty et al., 2018; Maillet et al., 2018). The total sample consisted of 186 adults from the University of North Carolina at Greensboro (UNCG) and surrounding community. Participants were paid up to $100 based on their level of completion in the three-part study, which included magnetic resonance imaging (MRI), daily-life experience-sampling, and laboratory assessments. Of the total sample, 172 participants completed both divergent thinking assessments; one participant was excluded as a multivariate outlier (Cooks Distance > 10), yielding a final sample of 171 (123 females, mean age = 22.63 years, SD = 6.29). All participants were right-handed with normal or corrected-to-normal vision, and they were not enrolled in the study if they reported a history of neurological disorder, cognitive disability, or medication and other drugs known to affect the central nervous system. The study was approved by the UNCG Institutional Review Board, and participants provided written informed consent prior to completing the study.

### Procedure

Participants completed a battery of tasks and questionnaires that measure different aspects of verbal creative ability (divergent thinking; novel metaphor production), real-world creative behavior (activities and achievements), and creative self-concept (self-efficacy and identity). Cognitive assessments were administered in a laboratory setting using MediaLab; questionnaires were administered both in the lab via MediaLab and online via Qualtrics.

#### Divergent thinking

Participants completed two trials of the AUT. The two trials (box and rope) were completed in a conventional testing environment on a computer running MediaLab (3 minutes of continuous idea generation). As in our prior work (Nusbaum et al., 2014), participants were instructed to “think creatively” while coming up with uses for the objects; notably, the instructions explicitly emphasized quality over quantity, as well as novelty over usefulness. Responses were subsequently scored for creative quality using the subjective scoring method (Benedek et al., 2013; Silvia et al., 2008). Four raters scored responses using a 1 (*not at all creative*) to 5 (*very creative*) scale. We provide task instructions and rater guidelines in the Supplemental Materials (also available via OSF; https://osf.io/vie7s/).

#### Creative behavior

We administered a battery of questionnaires to measure two facets of creative behavior: 1) creative activities (i.e., hobbies) and 2) creative achievements. Creative activities were assessed using the Biographical Inventory of Creative Behavior (BICB; Batey, 2007), which presents a list of 34 creative activities (e.g., making a website) and asks participants if they have participated in each activity within the past year (yes/no response). The Inventory of Creative Activities and Achievements (ICAA; Diedrich et al., 2018) includes two subscales that capture both creative activities/hobbies and higher-level accomplishments across eight domains of the arts and sciences. The Creative Achievement Questionnaire (CAQ; Carson, Peterson, & Higgins, 2005) assesses publicly-recognized creative achievements across ten creative domains.

#### Creative self-concept

The Short Scale of Creative Self (SSCS; Karwowski, 2014) assessed creative self-perceptions. The SSCS (11 items) captures two components of creative self-concept: creative self-efficacy (CSE) and creative personality identity (CPI). The CSE subscale measures the extent to which people perceive themselves as capable of solving creative challenges, such as “I am good at proposing original solutions to problems.” The CPI measures the extent to which creativity is a defining feature of the self-concept, such as “Being a creative person is important to me.”

#### Creative metaphor

As a further test of validation with a cognitive assessment of creativity with human ratings, we included two creative metaphor production prompts (Beaty & Silvia, 2013). Participants were presented with two open-ended prompts (i.e., common everyday experiences) and asked to produce novel metaphors to describe these experiences. One prompt asked participants, “Think of the most boring high-school or college class that you’ve ever had. What was it like to sit through?” Another prompt asked participants, “Think about the most disgusting thing you ever ate or drank. What was it like to eat or drink?” (Beaty & Silvia, 2013; Silvia & Beaty, 2012). Four raters scored the two metaphors using a 1 (*not at all creative*) to 5 (*very creative*) scale; the same four raters that scored the divergent thinking responses scored the metaphor responses.

#### Fluid intelligence

Past work indicates that fluid intelligence (Gf)—the ability to solve novel problems through reasoning—correlates positively with human creativity ratings on divergent thinking tests (Beaty, Silvia, Nusbaum, Jauk, & Benedek, 2014; Benedek, Jauk, Sommer, Arendasy, & Neubauer, 2014; Jauk et al., 2014). We thus included several measures of Gf to determine whether automated creativity ratings similarly relate to Gf, including: 1) the series completion task from Cattell’s Culture Fair Intelligence Test (Cattell & Cattell, 1973), which presents a row of boxes containing changing patterns and asks participants to choose the next image in the sequence based on the rule governing their change (13 items, 3 min); 2) the letter sets task (Ekstrom, French, Harman, & Dermen, 1976), which presents sequences of changing sets of letters and asks participants to choose the next letter set in the sequence (16 items, 4 min); and 3) the number series task (Thurstone, 1938), which presents sequences of changing sets of numbers and asks participants to choose the next number set in the sequence (15 items, 4.5 min).

#### Personality

We administered the 240 item NEO PI-R to assess the five major factors of personality (McCrae, Costa, & Martin, 2005). The full NEO includes six facet-level subscales for each personality factor, which were averaged to form composites for each of the five personality factors: neuroticism, extraversion, openness to experience, agreeableness, and conscientiousness. Participants were presented with a series of statements and asked to indicate their level of agreement using a five-point Likert scale (1 = *strongly disagree*, 5 = *strongly agree*).

### Semantic spaces

Five semantic spaces were selected based on the following criteria: 1) validity evidence showing associations between semantic distance and human judgments of semantic relatedness was available, 2) varied in the model used to compute word vectors, and 3) varied in the corpora used in the computational model. We used multiple computational models to build word vectors and various corpora because prior research indicates each model has idiosyncratic strengths and weaknesses in predicting human performance, with some models exhibiting advantages in predicting free association and others showing advantages in predicting human relatedness judgments (Mandera et al., 2017). Given the variety of methodologies employed to assess creativity, we reasoned that varied model selection would provide the highest generalizability and validity. Two semantic spaces were built using a neural network architecture, which uses a sliding window to move through the text corpora and tries to predict a central word from its surrounding context, similar to algorithms first developed in *word2vec* (Mikolov, Sutskever, Chen, Corrado, & Dean, 2013). These two continuous bag of words (CBOW) models have previously demonstrated robust associations with human judgments of relatedness, lexical decision speed, and free associations (Mandera et al., 2017). The first CBOW model is built on a concatenation of the ukwac web crawling corpus (~ 2 billion words) and the subtitle corpus (~ 385 million words). The second CBOW model was built on the subtitle corpus only. Each semantic space consisted of context window size of 12 words (six to the left and six to the right of the target words), 300 dimensions, and the most frequent 150,000 words (for more details, see Mandera et al., 2017).

The third semantic space was also built using CBOW but on a concatenation of the British National Corpus (~ 2 billion words), ukwac corpus, and the 2009 Wikipedia dump (~ 800 million tokens) using a context window size of 11 words, 400 dimensions, and the most frequent 300,000 words. This space also shows robust associations with human judgements of relatedness and was shown to be the best-performing model compared to multiple CBOW and LSA-based count models (Baroni, Dinu, & Kruszewski, 2014).

The fourth semantic space has the longest history and was built using LSA called TASA, from the Günther, Dudschig, and Kaup (2015) website and the lsacolorado.com interactive website. Termed a count model, it was built by computing the co-occurrence of words within documents, followed by a singular value decomposition on that sparse matrix. The corpus contained over 37,000 documents, including 92,393 different words, and was reduced to 300 dimensions. Primary text sources were middle and high school textbooks and literary works. This space demonstrates validity in its application to a creative word association task (Prabhakaran et al., 2014).

The fifth space was also built using a count model, but in contrast to LSA, it capitalizes on global information across the text using weighted least squares, called global vectors (GloVe; Pennington, Socher, & Manning, 2014). It was built on a concatenation of a 2014 Wikipedia dump and the Gigaword corpus, which contains numerous news publications from 2009–2010. The model was trained on ~ 6 billion tokens, with a final dimensionality of 300 and the top 400,000 words. GloVe has shown robust associations with human judgments of relatedness, comparable to other CBOW models (Pennington et al., 2014).

All five spaces can be used to compute the semantic distance between two words, where the cosine angle between the word vectors represents semantic similarity, and distance is then computed by subtracting this similarity from 1 (Beaty, Christensen, Benedek, Silvia, & Schacter, 2017; Green, 2016; Kenett, 2019; Prabhakaran et al., 2014). Semantic distance ranges from – 1 to 1, with higher scores indicating the two words are most distantly related ideas or concepts. The cosine was computed between word vectors using the LSAfun package of Günther et al. (2015) in R. However, when comparing words to phrases or phrases to phrases, the word vectors must be combined in some way to compute semantic distance. We describe this procedure in the Compositional Vector Models section below.

### Compositional vector models

All five of the above spaces are comprised of word vectors across a variable number of dimensions. When comparing texts that contain multiple words, a number of challenges arise; foremost being how to combine words vectors into a single vector for a comparison. Mitchell and Lapata (2008, 2010) investigated the strength of human relatedness judgments against various vector composition models of semantic distance. While additive models can perform adequately, multiplicative composition models performed best, even compared to more complex models like a weighted additive model. Consequently, most of our results are based on multiplicative vector composition models, where elementwise multiplication was used to combine vectors. However, the *SemDis* app gives users the option to choose additive or multiplicative models for semantic distance computations.

The other major challenge when dealing with phrases is how to clean text. Should all special characters be stripped? Should filler or stop words be removed? *SemDis* provides options for basic text cleaning, where only special characters and numbers are stripped, or to also remove filler or stop words. The stop words removed are based on the database from the *tm* R package (Feinerer, 2012). There is evidence that when applying latent semantic analysis to the AUT, removing stop words improves validity (Forthmann et al., 2018).

We provide a step-by-step tutorial with example data in the *SemDis* app with materials on OSF (https://osf.io/gz4fc/).

### Manual text preprocessing

Although *SemDis* provides preprocessing options, it does not include spellchecking, requiring users to manually spellcheck responses. This decision was made due to the impression of available spellchecking software and integration with the app; moreover, human intervention is often needed to resolve ambiguities in spellchecking. We recommend users employ spellchecking tools available in conventional software packages as they become available prior to uploading data files to *SemDis*. However, Johnson et al. (2019) did not employ spell checks and set misspelled words to missing data (the current default setting of *SemDis*). Combining misspelled words and words that the semantic model did not recognize resulted in a 4.1% loss of data. This minimal loss seems worth the labor savings if human raters instead had to perform spellchecking. In the current study, AUT responses were screened for misspelling and non-ambiguous spelling errors were corrected. As an additional optional step, the cue words (e.g., box and rope), as well as their plurals (e.g., boxes and ropes), were manually removed from responses to avoid potential bias of semantic distance values^1^.

### Analytic approach

Study 1 had two primary goals: 1) to compare semantic distance scores from several semantic spaces to human creativity ratings on the AUT and 2) to further validate these semantic distance scores against established creativity measures (e.g., creative behavior and achievement). Semantic distance scores, along with other creativity measures, were modeled as indicators of their respective latent variables, which allowed us to extract the common variance from each underlying factor. Latent variables were estimated using maximum likelihood estimation with robust standard errors in Mplus 8. The factor variances were fixed to 1, and the loadings for variables with less than three indicators were constrained to be equal (Kline, 2015).

In a first step, we conducted confirmatory factor analyses to model correlations between human creativity ratings and the five semantic distance variables. Next, we identified the best-performing semantic distance metric and probed its convergent validity in a series of structural equation models with the other creativity measures. To determine how human and automated creativity metrics differentially relate to creative activities and achievements, we modeled them as two separate latent variables (see below). All task variables were standardized prior to analysis. The standardized effects are presented in the *r* metric and can be interpreted using the conventional small (.10), medium (.30), and large (.50) guidelines (Cumming, 2013).

## Results

Table 1 presents zero-order correlations and descriptive statistics for creativity ratings and semantic distance models.

**Table 1:** Study 1 descriptive statistics and correlations of human ratings and multiplicative semantic distance models

	1	2	3	4	5	6	7	8	9	10	11	12	13	14	15	16	17	18	M	SD
1. b_r1	-																		1.45	0.32
2. b_r2	0.65	-																	1.39	0.41
3. b_r3	0.54	0.59	-																1.60	0.34
4. b_r4	0.73	0.74	0.73	-															1.91	0.47
5. b_cbu	0.46	0.48	0.38	0.45	-														0.90	0.07
6. b_cbs	0.33	0.37	0.46	0.39	0.76	-													0.94	0.05
7. b_cbw	0.43	0.45	0.36	0.39	0.84	0.69	-												0.93	0.05
8. b_tasa	0.30	0.27	0.23	0.22	0.36	0.34	0.41	-											0.98	0.03
9. b_glov	0.26	0.24	0.21	0.26	0.80	0.56	0.71	0.31	-										0.94	0.10
10. r_r1	0.29	0.38	0.37	0.43	0.18	0.10	0.10	-0.05	0.07	-									1.49	0.39
11. r_r2	0.27	0.39	0.39	0.41	0.17	0.14	0.10	0.08	0.08	0.65	-								1.33	0.38
12. r_r3	0.36	0.48	0.51	0.54	0.32	0.24	0.21	0.09	0.19	0.74	0.75	-							1.68	0.42
13. r_r4	0.37	0.43	0.48	0.51	0.32	0.22	0.22	0.11	0.21	0.74	0.76	0.87	-						1.74	0.53
14. r_cbu	0.36	0.40	0.36	0.49	0.43	0.35	0.41	0.11	0.36	0.32	0.37	0.57	0.54	-					0.86	0.09
15. r_cbs	0.31	0.41	0.39	0.45	0.39	0.33	0.40	0.13	0.30	0.34	0.38	0.54	0.54	0.84	-				0.91	0.07
16. r_cbw	0.35	0.42	0.37	0.49	0.49	0.43	0.46	0.16	0.39	0.32	0.34	0.53	0.51	0.90	0.87	-			0.90	0.07
17. r_tasa	0.22	0.34	0.28	0.34	0.28	0.19	0.17	0.03	0.23	0.32	0.33	0.53	0.46	0.66	0.62	0.60	-		0.95	0.05
18. r_glov	0.36	0.41	0.35	0.44	0.44	0.39	0.45	0.12	0.33	0.29	0.33	0.50	0.50	0.88	0.81	0.87	0.59	-	0.90	0.08

*Note*. *N* = 171; correlations greater than .16 are significant at *p*<.05; correlations greater than .2 are significant at *p* < .01. b_r1-b_r4 = AUT box, rater 1-rater 4; r_r1-r_r4 = AUT rope, rater 1-rater 4; r/b_glov = AUT rope/box, GloVe semantic distance; r/b_tasa = AUT rope/box, TASA semantic distance; r/b_cbw = AUT rope/box, continuous bag of words, Wiki concatenation, semantic distance; r/b_cbs = AUT rope/box, continuous bag of words, ukwac and subtitle corpus, semantic distance; r/b_cbu = AUT rope/box, continuous bag of words, subtitle corpus, semantic distance

### Predicting human creativity ratings

Our first set of analyses compared the relative prediction of human creativity ratings from additive vs. multiplicative compositional models of semantic distance. We began by conducting a confirmatory factor analysis to assess latent correlations between an additive semantic distance factor and human ratings on the two AUT items (box and rope): *χ*^2^ (132 *df*) 266.582, *p* < .001; CFI .927; RMSEA .077; SRMR .113. We found a moderate and negative correlation between the additive semantic distance factor and human ratings (*r* = -.37, *p* = .04), thus explaining only 14% variance in human creativity ratings.

Prior research that used additive composition models found that responses with higher word counts received a penalty in semantic distance, meaning lower semantic distance scores (Forthmann et al., 2018). Replicating this result, we found word count per response was negatively correlated with semantic distance scores (*r* = – 0.25). This is problematic, because higher word count responses (i.e., responses higher in elaboration) were rated by humans as being more creative (*r* = 0.41 between response word count and the mean creativity score for raters). Consequently, with humans giving higher ratings to longer responses, and semantic distance generating lower values, word count seems to explain the negative correlation between human rating and the additive semantic distance factor. Next, we test whether a multiplicative composition model can mitigate this issue.

**Fig. 1:**
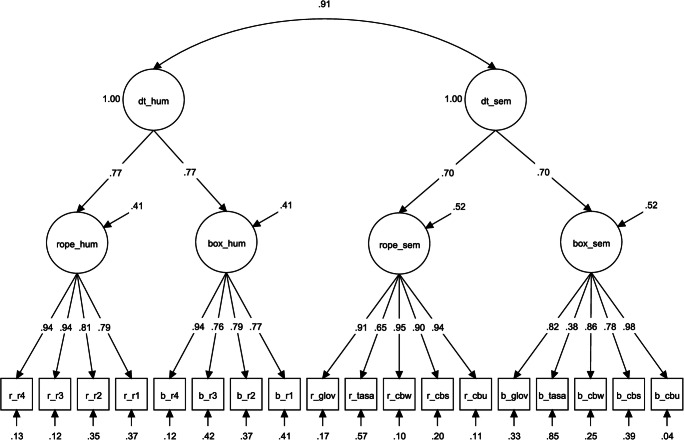
*Confirmatory factor analysis of human creativity ratings and multiplicative semantic distance for two AUT items. N* = 171. dt_hum = divergent thinking, human rating; dt_sem = divergent thinking, semantic distance; rope_hum = AUT rope, human rating; box_hum = AUT box, human rating; rope_sem = AUT rope, semantic distance; box_sem = AUT box, semantic distance; r_r1-r_r4 = AUT rope, rater 1-rater 4; ; b_r1-b_r4 = AUT box, rater 1-rater 4; r/b_glov = AUT rope/box, GloVe semantic distance; r/b_tasa = AUT rope/box, TASA semantic distance; r/b_cbw = AUT rope/box, continuous bag of words, Wiki concatenation, semantic distance; r/b_cbs = AUT rope/box, continuous bag of words, ukwac and subtitle corpus, semantic distance; r/b_cbu = AUT rope/box, continuous bag of words, subtitle corpus, semantic distance

**Fig. 2:**
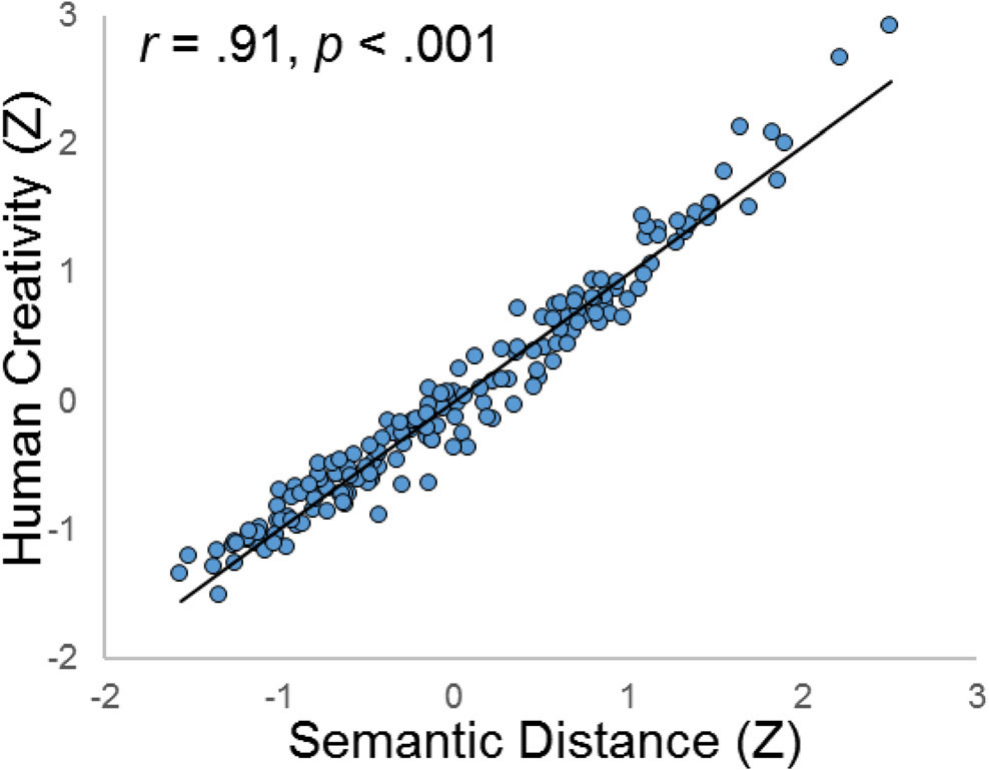
Scatterplot of the correlation between latent semantic distance and human creativity ratings in Study 1. Latent variable values are standardized for visualization. N = 171

We specified a model assessing the relationship between a multiplicative semantic distance model and human creativity ratings (Fig. 1). This model fit the data well: *χ*^2^ (132 *df*) 185.785, *p* < .001; CFI .970; RMSEA .049; SRMR .079. Results revealed a large correlation between latent semantic distance and human ratings: *r* = .91, *p* < .001 (Fig. 2). Thus, 83% of the variance in human ratings could be explained by a latent factor of five multiplicative semantic distance models. It is important to note this is much higher than the variance explained by the latent semantic factor derived from additive models.

In addition, the multiplicative composition model reversed the correlation between response word count and semantic distance (*r* = .47). For this model, responses with more elaboration now receive a boost in semantic distance. This is consistent with human creativity ratings, which also give a boost to more elaborate responses (*r* = .41), as noted above. This is a critical new finding because it shows a multiplicative model can substantially mitigate the elaboration bias demonstrated in prior research using semantic distance to capture creativity (Forthmann et al., 2018).

### Validation with external measures

Having found that multiplicative models outperform additive models in predicting human creativity ratings, we turned to further validate multiplicative models with a range of external creativity measures, spanning cognition (novel metaphor), behavior (creative achievement), and self-report (creative self-efficacy).

We began by specifying a CFA with the same latent semantic distance and human creativity variables, adding a higher-order novel metaphor factor comprised of two lower-order metaphor prompts, four raters per prompt (*χ*^2^ (293 *df*) 362.249, *p* < .001; CFI .973; RMSEA .037; SRMR .070). The model yielded significant correlations between creative metaphor and both AUT creativity (*r* = .49, *p* = .001) and AUT semantic distance (*r* = .41, *p* = .005), indicating converging validity of semantic distance at the cognitive level.

Our next analysis focused on creative behavior. We specified a latent variable comprised of the four creative behavior scales, along with the same AUT creativity and AUT semantic distance variables (*χ*^2^ (204 *df*) 276.402, *p* < .001; CFI .967; RMSEA .046; SRMR .078). Consistent with past work, creative behavior correlated significantly with AUT creativity (*r* = .43, *p* < .001). The model also showed a small effect for AUT semantic distance (*r* = .21, *p* = .04).

Next, we assessed effects of creative self-efficacy, specifying a latent variable comprised of its two lower-order facets, along with AUT creativity and AUT semantic distance (*χ*^2^ (166 *df*) 225.198, *p* < .001; CFI .971; RMSEA .046; SRMR .078). The model showed significant correlations between creative self-efficacy and both AUT creativity (*r* = .36, *p* < .001) and AUT semantic distance (*r* = .32, *p* = .002), replicating prior work on AUT creativity and providing further converging validity for semantic distance at the level of creative personality.

Finally, we examined effects of fluid intelligence and personality. We first specified a model with fluid intelligence, AUT creativity, and AUT semantic distance (*χ*^2^ (184 *df*) 246.041, *p* < .001; CFI .970; RMSEA .044; SRMR .073). Fluid intelligence correlated significantly with AUT creativity (*r* = .36, *p* = .003), consistent with past work, but it showed a small and nonsignificant effect on AUT semantic distance (*r* = .10, *p* = .39). Regarding personality, we specified a model with the five factors of personality correlating with the two AUT variables (*χ*^2^ (222 *df*) 357.001, *p* < .001; CFI .938; RMSEA .060; SRMR .080) and found that only openness correlated with AUT creativity (*r* = .30, *p* < .001) but not AUT semantic distance (*r* = .03, *p* = .77); no other personality factors showed significant effects on AUT creativity or semantic distance.

## Study 2

Study 1 provided preliminary evidence for the validity of semantic distance in predicting human judgements of creativity on the AUT. We found that a latent variable comprised of five semantic distance metrics strongly correlated with human subjective creativity ratings. Semantic distance scores also correlated positively with cognitive and self-report measures related to creativity (metaphor production, creative self-efficacy) but not to other cognitive and personality factors (fluid intelligence and openness). In Study 2, we aimed to replicate a subset of findings from Study 1, using the same AUT items from a previously published dataset (Silvia, Nusbaum, & Beaty, 2017). To this end, we employed the same approach to computing semantic distance from Study 1, and we reanalyzed subjective creativity scores obtained from the original study. We hypothesized that the latent semantic distance variable would again predict human judgements of creativity on the AUT. Notably, Silvia et al. (2017) found that human creativity ratings on the AUT did not significantly correlate with creative behavior, so it remains unclear whether semantic distance scores could predict creative behavior in this sample. We also again tested whether semantic distance correlated with openness, which was not the case in Study 1, but was reported by Prabhakaran et al. (2014) in their study with the noun-verb task. Furthermore, Study 2 sought to extend Study 1 by examining whether semantic distance scores also relate to participants’ self-ratings of creativity on the AUT.

## Method

### Participants

Data for this study were reanalyzed from Silvia et al. (2017), which aimed to validate the old/new scoring method for divergent thinking. The final sample included 142 adults from the University of North Carolina at Greensboro (UNCG; mean age = 19.22, SD = 3.07; 70% female). The study was approved by the UNCG IRB and participants received credit toward a voluntary research option for their time.

### Procedure

Participants completed a series of divergent thinking tasks (AUT) and self-report measures related to creativity (creative behavior and openness). All measures were administered on laboratory computers running MediaLab.

#### Divergent thinking

As in Study 1, participants completed two AUT items: box and rope. Likewise, they were asked to “think creatively” while coming up with their responses. Participants were given three minutes to type their responses (note that Study 1 had a 2-min time limit). AUT responses were again scored using the subjective scoring method (Silvia et al., 2008). Three trained raters scored each response using a 1 (*not at all creative*) to 5 (*very creative*) scale. Like Study 1, raters were blind to participants’ identity and the serial order of their responses.

After completing the two AUT items, participants were shown their responses and asked to rate the creativity of each response on the same five-point scale. Specifically, they were asked to indicate “how creative, in your opinion, each idea is.” Responses were presented in the order in which they were produced by each participant.

#### Creative behavior

Participants completed two of the same measures of creative behavior from Study 1. To assess creative activities, they completed the BICB (Batey, 2007); to assess creative achievements, they completed the CAQ (Carson et al., 2005).

#### Openness to experience

Personality was measured using the HEXACO (Lee & Ashton, 2004), which assesses four facets of openness to experience: aesthetic appreciation, unconventionality, intuitiveness, and creativity. Participants responded to each openness item using a five-point scale (1 = *strongly disagree*, 5 = *strongly agree*).

## Results

Table 2 presents zero-order correlations and descriptive statistics for creativity ratings and semantic distance models. Consistent with Study 1, we found that the five multiplicative compositional models correlated positively and variably with individual human raters, with the largest correlations again observed for CBOW models.

**Table 2:** Study 2 descriptive statistics and correlations of human ratings and multiplicative semantic distance models

	1	2	3	4	5	6	7	8	9	10	11	12	13	14	15	16	M	SD
1. b_r1	-																2.17	0.49
2. b_r2	0.59	-															1.69	0.55
3. b_r3	0.73	0.73	-														1.74	0.48
4. b_cbu	0.35	0.54	0.42	-													0.91	0.07
5. b_cbs	0.15	0.37	0.30	0.77	-												0.95	0.05
6. b_cbw	0.17	0.41	0.40	0.77	0.74	-											0.95	0.05
7. b_tasa	– 0.06	0.06	0.03	0.32	0.39	0.28	-										0.98	0.03
8. b_glov	0.18	0.31	0.29	0.66	0.56	0.53	0.23	-									0.94	0.09
9. r_r1	0.40	0.56	0.44	0.36	0.26	0.26	– 0.10	0.24	-								2.07	0.51
10. r_r2	0.42	0.63	0.53	0.46	0.28	0.32	– 0.05	0.35	0.75	-							1.61	0.54
11. r_r3	0.30	0.57	0.43	0.30	0.24	0.23	– 0.16	0.25	0.76	0.74	-						1.50	0.37
12. r_cbu	0.13	0.40	0.30	0.44	0.40	0.42	0.03	0.29	0.40	0.55	0.40	-					0.88	0.09
13. r_cbs	0.13	0.34	0.27	0.44	0.38	0.42	0.08	0.38	0.31	0.47	0.38	0.82	-				0.93	0.07
14. r_cbw	0.16	0.38	0.28	0.50	0.42	0.47	0.12	0.42	0.31	0.48	0.34	0.84	0.90	-			0.92	0.07
15. r_tasa	0.18	0.30	0.20	0.41	0.40	0.33	0.12	0.32	0.32	0.42	0.40	0.58	0.67	0.66	-		0.96	0.05
16. r_glov	0.21	0.38	0.33	0.48	0.46	0.42	0.11	0.42	0.31	0.40	0.30	0.84	0.77	0.79	0.61	-	0.92	0.08

*Note*. *N* = 142; correlations greater than .17 are significant at *p* < .05; correlations greater than .22 are significant at *p* < .01. b_r1-b_r3 = AUT box, rater 1-rater 4; r_r1-r_r3 = AUT rope, rater 1-rater 4; r/b_glov = AUT rope/box, GloVe semantic distance; r/b_tasa = AUT rope/box, TASA semantic distance; r/b_cbw = AUT rope/box, continuous bag of words, Wiki concatenation, semantic distance; r/b_cbs = AUT rope/box, continuous bag of words, ukwac and subtitle corpus, semantic distance; r/b_cbu = AUT rope/box, continuous bag of words, subtitle corpus, semantic distance

**Fig. 3:**
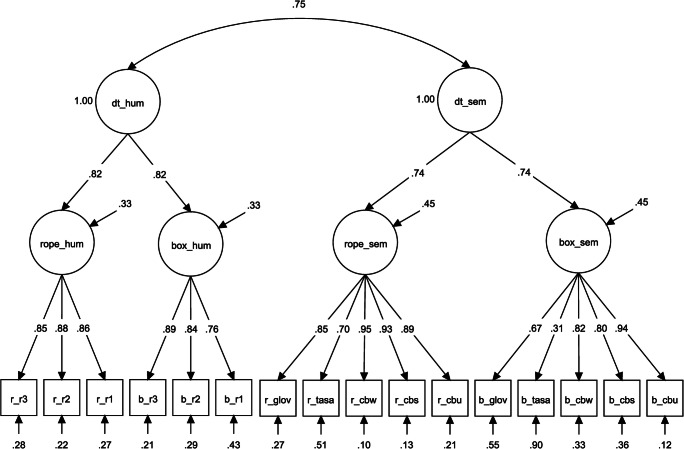
Confirmatory factor analysis of human creativity ratings and multiplicative semantic distance for two AUT items. *N* = 142. dt_hum = divergent thinking, human rating; dt_sem = divergent thinking, semantic distance; rope_hum = AUT rope, human rating; box_hum = AUT box, human rating; rope_sem = AUT rope, semantic distance; box_sem = AUT box, semantic distance; r_r1-r_r3 = AUT rope, rater 1-rater 3; ; b_r1-b_r3 = AUT box, rater 1-rater 3; r/b_glov = AUT rope/box, GloVe semantic distance; r/b_tasa = AUT rope/box, TASA semantic distance; r/b_cbw = AUT rope/box, continuous bag of words, Wiki concatenation, semantic distance; r/b_cbs = AUT rope/box, continuous bag of words, ukwac and subtitle corpus, semantic distance; r/b_cbu = AUT rope/box, continuous bag of words, subtitle corpus, semantic distance

**Fig. 4:**
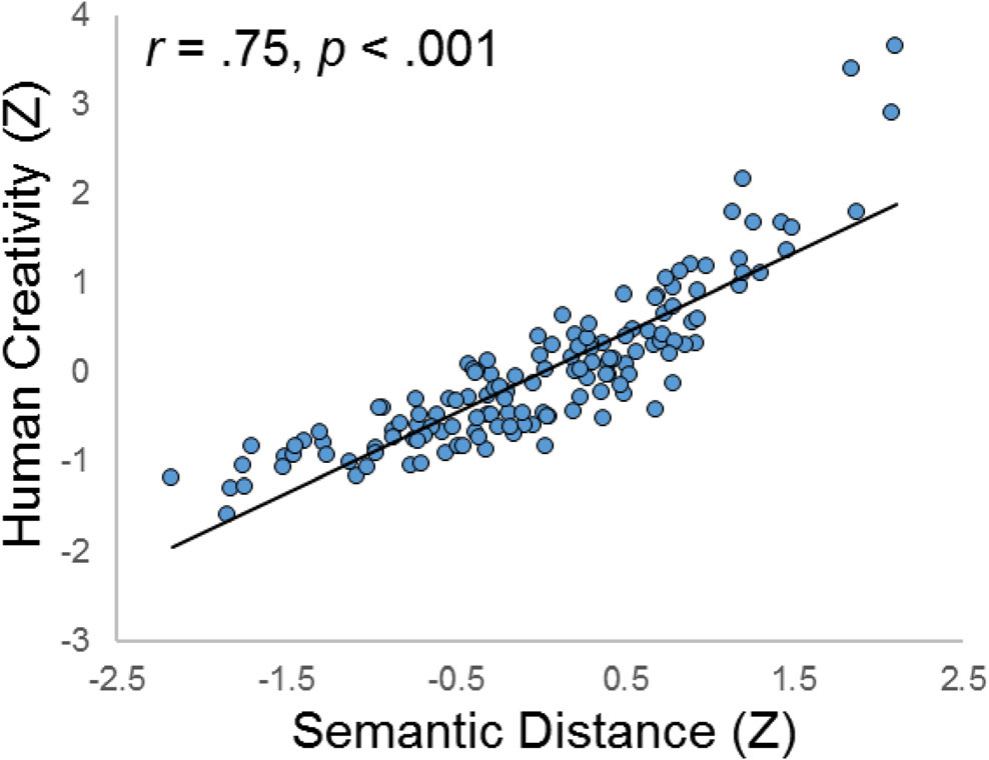
Scatterplot of the correlation between latent semantic distance and human creativity ratings in Study 2. Latent variable values are standardized for visualization. *N* = 142

We began by specifying a CFA with two latent variables—multiplicative semantic distance and human creativity ratings—using the same model specification as in Study 1: *χ*^2^ (101 *df*) 199.483, *p* < .001; CFI .924; RMSEA .083; SRMR .070. Figure 3 depicts the measurement model. Similar to Study 1, all semantic models loaded highly onto their respective latent variable, with the highest loadings from CBOW models. Moreover, consistent with Study 1, we found a large positive correlation between human creativity ratings and latent semantic distance: *r* = .75, *p* < .001 (Fig. 4). Thus, approximately half of the variance in human ratings could be explained by the common variance extracted from five semantic distance models.

Next, we turned to test whether semantic distance similarly predicts participants’ own self-assessments of their idea’s creativity. We thus specified a within-person, multilevel structural equation model, with latent semantic distance scores predicting self-ratings of creativity for each AUT item separately. For the *box* model (*χ*^2^ (9 *df*) 52.957, *p* < .001; CFI .984; RMSEA .064; SRMR .025), we found that latent semantic distance scores significantly predicted the self-ratings (unstandardized *b* = 1.03, SE = .42, *p* = 01): as semantic distance scores increased, participants rated their ideas as more creative. For the *rope* model (*χ*^2^ (9 *df*) 108.672, *p* < .001; CFI .983; RMSEA .097; SRMR .027), we also found a significant linear effect (unstandardized *b* = 1.71, *p* < .001), indicating that multiplicative semantic distance models track participants’ own assessment of their idea’s creativity.

### Validation with external measures

The external validation analysis assessed whether latent semantic distance and human creativity ratings relate to creative behavior and openness to experience. Creative behavior was modeled as a latent variable comprised of everyday hobbies (BICB) and real-world achievements (CAQ), along with AUT creativity and AUT semantic distance (*χ*^2^ (131 *df*) 243.750, *p* < .001; CFI .920; RMSEA .078; SRMR .069). Creative behavior did not significantly predict AUT creativity (*r* = .16, *p* = .09) or AUT semantic distance (*r* = .03, *p* = .84). Regarding openness, a model with latent openness and the two AUT variables (*χ*^2^ (165 *df*) 300.830, *p* < .001; CFI .913; RMSEA .076; SRMR .070) showed a significant correlation between openness and both AUT creativity (*r* = .48, *p* < .001) and AUT semantic distance (*r* = .24, *p* = .02).

## Study 3

Study 2 replicated the latent correlation between human creativity ratings and semantic distance ratings: a latent variable comprised of semantic distance values from five multiplicative compositional models strongly predicted human creativity ratings on the AUT. We also found that semantic distance relates to participants’ self-ratings of creativity, providing further evidence that semantic distance captures variance associated with creativity. Notably, Study 2 found that AUT semantic distance did not correlate with creative behavior; however, the same pattern was found for human creativity ratings on the AUT in Study 2, suggesting that, in this study, performance on the AUT—assessed via semantic distance and human ratings—did not capture variance associated with creative behavior. On the other hand, we found that, contrary to Study 1, openness significantly predicted AUT semantic distance, consistent with the verb generation study of Prabhakaran et al. (2014).

In Study 3, we sought to replicate and extend the findings of our first two studies. To this end, we reanalyzed data using a new and more commonly used AUT item (i.e., brick). We again tested whether semantic distance values correlated with human creativity ratings and other measures associated with creativity (i.e., openness and metaphor production); we also reassessed the relation between semantic AUT semantic distance and fluid intelligence. Furthermore, we tested an established experimental effect in the divergent thinking literature known as the *serial order effect*, the tendency for ideas to be rated as more original over time (Acar, Abdulla Alabbasi, Runco, & Beketayev, 2019; Beaty & Silvia, 2012; Christensen, Guilford, & Wilson, 1957; Hass & Beaty, 2018). Although prior work has reported serial order effects on the AUT with LSA (Hass, 2017b), we sought to replicate this effect and extend it by using a broader range of compositional semantic models.

## Method

### Participants

Data for this study were reanalyzed from Beaty and Silvia (2012) and Silvia and Beaty (2012), which used the same dataset. The final sample included 133 adults from the University of North Carolina at Greensboro (UNCG; mean age = 19.60, SD = 3.20; 69% female). The study was approved by the UNCG IRB and participants received credit toward a voluntary research option for their time.

### Procedure

#### Divergent thinking

Participants completed an extended AUT to assess temporal trends in idea generation (see Beaty & Silvia, 2012). They were given 10 min to continually generate uses for a brick. Note that the task duration was considerably longer than previous studies due to the temporal focus of Beaty and Silvia (2012). Each response was time-stamped to model serial order effects.

#### Personality

The NEO PI-R was administered to assess the five major factors of personality: neuroticism, extraversion, openness, agreeableness, and conscientiousness (McCrae et al., 2005). Each of the five factors was measured with two items (60 items total). Participants used a five-point scale to indicate their level of agreement with each item.

#### Creative metaphor

Participants completed the same creative metaphor prompts from Study 1 (i.e., ‘boring class’ and ‘gross food’; see Silvia & Beaty, 2012). As in Study 1, metaphor responses were scored for creative quality using the subjective scoring method (Silvia et al., 2008).

#### Fluid intelligence

Six nonverbal measures of fluid intelligence were administered: 1) a short version of the Ravens Advanced Progressive Matrices (18 items, 12 min); 2) a paper folder task (ten items, 3 min; Ekstrom et al., 1976); 3) a letter sets task (16 items, 4 min; Ekstrom et al., 1976); 4) the matrices task from the Cattell Culture Fair Intelligence Test (CFIT; 13 items, 3 min; Cattell & Cattell, 1961/2008); 5) the series task from the CFIT (13 items, 3 min); and 6) a number series ask (15 items, 4.5 min; (Thurstone, 1938).

## Results

Table 3 presents zero-order correlations and descriptive statistics for creativity ratings and semantic distance models.

**Table 3:** Study 3 descriptive statistics and correlations of human ratings and multiplicative semantic distance models

	1	2	3	4	5	6	7	8	M	SD
1. br_r1	-								1.43	0.36
2. br_r2	0.75								1.30	0.29
3. br_r3	0.71	0.78	-						1.90	0.48
4. br_cbu	0.38	0.30	0.48	-					0.94	0.05
5. br_cbs	0.39	0.34	0.47	0.76	-				0.96	0.04
6. br_cbw	0.33	0.31	0.42	0.80	0.82	-			0.97	0.04
7. br_tasa	0.21	0.15	0.25	0.45	0.52	0.58	-		0.99	0.03
8. br_glov	0.31	0.25	0.30	0.78	0.69	0.77	0.54	-	0.98	0.06

*Note*. *N* = 133; correlations greater than .18 are significant at *p* < .05; correlations greater than .23 are significant at *p* < .01. br_r1-br_r3 = AUT brick, rater 1-rater 3; br_glov = AUT brick, GloVe semantic distance; br_tasa = AUT brick, TASA semantic distance; br_cbw = AUT brick, continuous bag of words, Wiki concatenation, semantic distance; br_cbs = AUT brick, continuous bag of words, ukwac and subtitle corpus, semantic distance; br_cbu = AUT brick, continuous bag of words, subtitle corpus, semantic distance

### Predicting human creativity ratings

Our first analysis assessed the association between human creativity ratings and latent semantic distance. We thus specified a CFA with these two latent variables: *χ*^2^ (19 *df*) 50.090, *p* < .001; CFI .951; RMSEA .111; SRMR .046. The model yielded a positive and moderately large latent correlation between semantic distance and human ratings: *r* = .45, *p* < .001; the magnitude of this correlation is comparable to the magnitude of effects for single AUT items from Study 1 and 2.

### Semantic distance and serial order

Next, we examined whether semantic distance scores showed a serial order effect, i.e., a tendency for responses to become more original over time. To this end, we specified a within-person regression model, with time predicting latent semantic distance; factor loadings were constrained to be equal for model convergence: *χ*^2^ (13 *df*) 286.036, *p* < .001; CFI .976; RMSEA .108; SRMR .090. This model showed a significant effect of time on semantic distance: unstandardized *b* = .05, SE = .01, *p* < .001: as time increased from 0 to 10 min, so did the semantic distance of AUT responses, demonstrating a serial order effect.

### Validating with external measures

Our first external validation analysis assessed correlations between the Big 5 personality factors, AUT human ratings, and semantic distance scores: *χ*^2^ (59 *df*) 130.418, *p* < .001; CFI .909; RMSEA .095; SRMR .075. Consistent with past work, of the five personality factors, only openness to experience correlated significantly with human creativity ratings: *r* = .57, *p* < .001. Replicating Study 2, the model also showed a significant correlation between openness and semantic distance scores: *r* = .19, *p* = .007.

Next, we assessed correlations between creative metaphor, human ratings, and semantic distance: *χ*^2^ (73 *df*) 141.225, *p* < .001; CFI .922; RMSEA .084; SRMR .059. Replicating Study 1, we found that creative metaphor positively correlated with both human ratings (*r* = .39, *p* = .005) and semantic distance scores (*r* = .20, *p* = .05).

Regarding fluid intelligence, Beaty and Silvia (2012) previously reported a positive relation between fluid intelligence and AUT creativity ratings (*r* = .26). We specified a model with fluid intelligence and latent semantic distance (*χ*^2^ (43 *df*) 56.795, *p* = .078; CFI .978; RMSEA .049; SRMR .047). We found that fluid intelligence correlated with AUT semantic distance to approximately the same degree as reported for AUT creativity in Beaty and Silvia (2012): *r* = .24, *p* = .01.

## Study 4

Study 3 replicated and extended findings from Studies 1 and 2. Using a new and more commonly used AUT object (i.e., brick), we found that latent semantic distance factor again predicted human creativity ratings, indicating that the relation between creativity ratings and semantic distance is not item-dependent. We also found that semantic distance was sensitive to an established experimental effect in the creativity literature known as the serial order effect: as time increased, the semantic distance of responses also increased, consistent with findings for human ratings. Regarding external measures, semantic distance significantly related to openness and fluid intelligence, partially replicating Studies 1 and 2.

In Study 4, we sought to extend our application of semantic distance beyond the AUT to a word association task employed in the creativity literature: the verb generation task (Prabhakaran et al., 2014). The verb generation task presents a series of nouns and asks participants to generate either common or creative verbs that can be related to the nouns; responses have commonly been assessed via LSA (Green, 2016). Here, we reanalyze verb generation data from two studies conducted by Heinen and Johnson (2018) that includes human ratings on multiple dimensions: novelty, creativity, and appropriateness. Heinen and Johnson (2018) previously reported moderate to large correlations between these dimensions and semantic distance computed via LSA. Here, we aim to test whether a latent semantic distance factor extracted from the five semantic models improves the prediction of human creativity compared to average scores computed via LSA.

## Method

### Participants

We reanalyzed data from two samples of participants from Heinen and Johnson (2018). Sample 1 (*n* = 62, 39 women, mean age = 37 years, age range: 20–60) and Sample 2 (*n* = 56, women = 30, mean age = 37, age range = 20-69) were recruited from Amazon Mechanical Turk (M-Turk). All participants were compensated $.50 to complete the half-hour study.

### Materials

Sample 1 and Sample 2 included the same stimulus set, which consisted of 60 common nouns taken from the Appendix of Prabhakaran et al. (2014). The nouns varied on level of constraint, or the extent to which they tended to yield a restricted range of response (e.g., for the noun ‘scissors’, most participants produce the verb ‘cut’; Heinen & Johnson, 2018; Prabhakaran et al., 2014). A goal of Heinen and Johnson (2018) was to test whether varying instructional cues to generate common, random, or creative verbs impacted semantic distance. The authors thus created three lists of 20 nouns that were matched on constraint (see Heinen & Johnson, 2018, for details on the stimulus set).

### Procedure

Sample 1 was a within-subjects design and Sample 2 was a between-subjects design. In Sample 1, all participants received the three cued instructions in a fixed order: common, random, and creative, respectively (cf., Harrington, 1975; Wilken, Forthmann, & Holling, 2019). In Sample 2, participants were randomly assigned to one of four conditions: common, random, specific creative (additional creativity instruction), and nonspecific creative (minimal creativity instruction; see Heinen & Johnson, 2018). For the present analysis, we only included the creative trials in both samples (1 creative condition in Study 1, 2 creative conditions in Study 2), due to our goal of validating semantic distance in the assessment of creativity (not randomness or commonness). In both samples, after responding to demographic questions, participants completed a self-paced verb generation task. Each trial presented a noun on the screen; participants were asked to think of a verb (based on instruction condition) and to advance to the next slide as soon as their response was in mind. Then, they were instructed to type their verb response into a textbox. Participants were given a break after 20 trials, during which they received a new instruction set (Sample 1; within-subjects) or a brief reminder of how to respond in each instruction condition (Sample 2; between-subjects).

### Subjective rating

Two trained raters blind to experimental condition coded responses from Sample 1 and Sample 2 on three dimensions: creativity, novelty, and appropriateness. The purpose of this coding scheme was to determine whether LSA was sensitive to variation in cued instruction (i.e., creative, random, and common) and whether LSA values correlate with human ratings. One rater coded 31 of the 60 nouns, another rater coded 27 nouns, and both raters coded two nouns to assess interrater reliability. The ratings were completed using the same five-point scale (1 = *not* X to 5 = *definitely* X), with X corresponding to creative, novel, or related. Similar to the AUT, responses were coded using a subjective scoring method that followed the guidance and definitions of the consensual assessment technique (Amabile, 1983). Specifically, appropriateness/relatedness was defined as the extent to which a response was “comprehensible, understandable, and accessible”; novelty was defined as “originality or newness…a novel response can be completely unrelated to the noun”; and creativity was defined as a combination of novelty and appropriateness (cf., Diedrich et al., 2015), with the addition of “cleverness” and “non-obviousness.” In both samples, raters coded creativity first, followed by novelty and appropriateness.

### Latent factor extraction

One goal of this study was to test how a latent factor score, comprised of the five semantic spaces, relates to human ratings of novelty, creativity, and appropriateness. Notably, this approach extends our first three studies by computing factor scores at the trial-level, not the individual subject level. We built this capability into the *SemDis* platform, allowing users to leverage the power of SEM, regardless of their level of expertise. The latent variable, derived from the five semantic models, was modeled at the item-level in the *lavaan* R package using the *cfa()* function and factor scores were computed in the *lavPredict()* function (Rosseel, 2012).

## Results

### Sample 1

Table 4 presents zero-order correlations and descriptive statistics for creativity ratings and semantic distance models. A total of 1,240 verb responses were included in the analysis. The response set was cleaned following the procedure described in Heinen and Johnson (2018), i.e., removal of unambiguous spelling errors, additional words, and ending suffixes. Interrater reliability of the two nouns assessed by the two raters (*n* = 115 verb responses) showed strong interrater agreement across the three scoring dimensions: creativity (*r* = .72), novelty *(r* = .74), and appropriateness (*r* = .75).

**Table 4:** Study 4/Sample 1 descriptive statistics and correlations of human ratings and multiplicative semantic distance models

	1	2	3	4	5	6	7	8	9	M	SD
1. creativity	-									2.20	0.40
2. novelty	0.80	-								2.80	0.68
3. appropriateness	– 0.19	– 0.63	-							3.53	0.56
4. cbu	0.55	0.78	– 0.76	-						0.78	0.05
5. cbs	0.35	0.63	– 0.85	0.90	-					0.85	0.05
6. cbw	0.32	0.55	– 0.77	0.90	0.93	-				0.87	0.04
7. tasa	0.54	0.72	– 0.76	0.85	0.84	0.81	-			0.92	0.05
8. glov	0.61	0.84	– 0.75	0.94	0.86	0.85	0.82	-		0.83	0.07
9. semdis_factor	0.49	0.73	– 0.81	0.98	0.96	0.96	0.88	0.95	-	0.00	0.05

*Note*. *N* = 62; correlations greater than .26 are significant at *p* < .05; correlations greater than .33 are significant at *p* < .01. GloVe semantic distance; tasa = TASA semantic distance; cbw = continuous bag of words, Wiki concatenation, semantic distance; cbs = continuous bag of words, ukwac and subtitle corpus, semantic distance; cbu = continuous bag of words, subtitle corpus, semantic distance

#### Correlations between human ratings and semantic distance

**Fig. 5:**
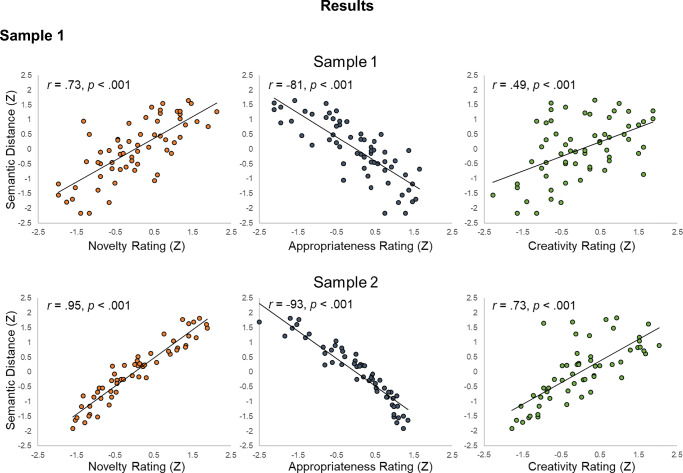
Scatterplots of the correlations between latent semantic distance and novelty, appropriateness, and creativity ratings in Study 4. Latent variable values are standardized for visualization. Sample 1, *N* = 62; Sample 2, *N* = 56

We began by computing Pearson correlations between mean human ratings (creativity, novelty, appropriateness) and semantic distance (latent factor and five semantic models; see Table 4 and Fig. 5). Novelty and appropriateness were strongly negatively correlated (*r* = – .63); novelty and creativity were strongly positively correlated (*r* = .80); and creativity and appropriateness were negatively (but not significantly) correlated (*r* = – .19, *p* = .14).

Next, we computed correlations between human ratings and semantic distance for each semantic model. Regarding creativity, the five semantic spaces showed comparable but variable correlations with creativity ratings, with the highest correlations found for GloVe (*r* = .61) and cbowukwac (*r* = .55). The latent semantic distance factor showed a moderately large correlation with human creativity ratings (*r* = .49). Regarding novelty, we found larger correlations with semantic distance, with GloVe again showing the largest effect size (*r* = .84) followed by cbowukwac (*r* = .78); the latent factor correlated with human novelty ratings to a similar but attenuated degree as the individual models, *r* = .73, likely due to the lower correlations with other models. Regarding appropriateness, we found similarly large but negative correlations with semantic distance (e.g., GloVe *r* = – .75), consistent with the inverse relation between novelty and appropriateness (see Table 4); the latent factor correlated similarly with appropriateness, *r* = –.81.

### Sample 2

Table 5 presents zero-order correlations and descriptive statistics for creativity ratings and semantic distance models. A total of 3,360 verb responses were included in the analysis. The same preprocessing procedure was applied as in Study 1 (e.g., removal of unambiguous spelling errors). Inter-rater reliability was generally high but varied across the four instruction conditions and dependent measures (see Heinen & Johnson, 2018, Appendix B).

**Table 5:** Study 4/Sample 2 descriptive statistics and correlations of human ratings and multiplicative semantic distance models

	1	2	3	4	5	6	7	8	9	M	SD
1. creativity	-									1.90	0.42
2. novelty	0.83	-								2.48	0.76
3. appropriateness	– 0.55	– 0.91	-							3.83	0.69
4. cbu	0.76	0.96	– 0.92	-						0.74	0.07
5. cbs	0.66	0.91	– 0.94	0.98	-					0.81	0.06
6. cbw	0.66	0.89	– 0.91	0.97	0.98	-				0.84	0.04
7. tasa	0.75	0.92	– 0.88	0.96	0.95	0.95	-			0.88	0.07
8. glov	0.78	0.97	– 0.91	0.98	0.94	0.94	0.94	-		0.79	0.08
9. semdis_factor	0.73	0.95	– 0.93	1.00	0.99	0.98	0.97	0.98	-	-0.01	0.06

*Note*. *N* = 56; all correlations are significant at *p* < .001. glov = GloVe semantic distance; tasa = TASA semantic distance; cbw = continuous bag of words, Wiki concatenation, semantic distance; cbs = continuous bag of words, ukwac and subtitle corpus, semantic distance; cbu = continuous bag of words, subtitle corpus, semantic distance

#### Correlations between human ratings and semantic distance

We computed Pearson correlations between the three human ratings (creativity, novelty, appropriateness) and semantic distance (latent factor and five semantic models; see Table 5 and Fig. 5). Creativity and novelty ratings were positively correlated (*r* = .83); novelty and appropriateness were strongly negatively correlated (*r* = – .91); and creativity and appropriateness were negatively correlated (*r* = – .55). The pattern of human rating correlations is thus comparable to Sample 1.

Next, we assessed correlations between the human creativity ratings and semantic distance. Replicating Sample 1 (and consistent with results reported in Heinen & Johnson, 2018), the five semantic models showed large correlations with creativity ratings; the largest correlations were found between creativity ratings and GloVe (*r* = .78), but similarly large correlations were seen for cbowukwac (*r* = .76) and TASA (*r* = .75). The latent semantic distance variable showed a large effect consistent with the correlational pattern of the five individual models (*r* = .73; Fig. 5).

Regarding novelty, all five semantic models showed a near perfect correlation with novelty ratings: GloVe (*r* = .97), cbowukwac (*r* = .96), cbowsubs (*r* = .91), TASA (*r* = .92), and cbowBNC (*r* = .89). These large effect sizes were reflected in the latent factor score: *r* = .95.

Regarding appropriateness, a similar pattern emerged, albeit in the opposite direction. Human ratings of appropriateness were strongly negatively correlated with semantic distance: GloVe (*r* = – .91), cbowukwac (*r* = – .92), cbowsubs (*r* = – .94), TASA (*r* = – .88), and cbowBNC (*r* = – .91); the latent semantic distance factor was comparable in magnitude (*r* = – .93).

## Study 5

Study 4 extended the recent word association work of Heinen and Johnson (2018) examining the correspondence between semantic distance and human ratings of creativity, novelty, and appropriateness. In two datasets, using data from a noun-verb association task, we found that the five semantic models showed the strongest associations with human ratings of novelty and appropriateness—with correlations approaching unity—and moderate associations with human rating of creativity. These results extend the current application of semantic distance in creativity assessment using the verb generation task, which is increasingly used in creativity research.

In Study 5, we sought to replicate and extend the findings using a second word association task requiring multiple responses. To this end, we reanalyzed data from Johnson et al. (2019), who employed a noun association task to study the “idea diversity” of responses, i.e., the extent to which responses semantically diverge from each other, rather than diverge from the response cue—similar to the “flexibility” metric in divergent thinking tasks. In these studies, participants were asked to generate either two or four creative words in response to a given noun. Novelty was assessed by computing the average semantic distance between the noun prompt and participant’s creative responses. To assess whether human ratings of creativity correspond to semantic distance, we obtained creativity ratings from three independent raters. This approach allowed us to test the relative performance of five semantic models, along with the latent factor score, in predicting human creativity ratings on a newly developed assessment of creative association making.

## Method

### Participants

We reanalyzed response data from the “any” condition in Study 2 of Johnson et al. (2019). Participants (*n* = 58, 57% women, mean age = 38 years, age range: 18–82) were recruited from Amazon Mechanical Turk (MTurk) and were compensated $1.50 to complete the half-hour study.

### Materials

In their original study, Johnson et al. (2019) designed a task to assess idea diversity, i.e., the conceptual variance between individual ideas, akin to flexibility in divergent thinking assessment. Participants completed a new word association task—the Corpus-based Assessment of Novelty and Diversity (C-BAND)—that presents a series of nouns and asks participants to generate four noun responses. The nouns could be of any type with the exception of proper nouns. Participants were asked to “think creatively” and come up with associations that could be creatively linked to the given noun.

### Procedure

To assess the effect of instruction on idea diversity, Johnson et al. (2019) randomly assigned participants to three conditions. We included participants from the “any” condition because the instructions are closest to those commonly given to participants in creativity studies (see Supplemental Materials). Following demographic questions and task instruction, participants completed one practice trial with the cue word *dog*. Then, they completed eight experimental trials of the C-BAND. Each trial presented a noun and asked participants to generate four associations with no time limit.

### Subjective rating

The aim of this study was to validate semantic distance against human creativity ratings using a new word association task. We therefore obtained creativity ratings from three MTurk workers who were thoroughly briefed on the scoring protocol (see Supplemental Materials). MTurk workers have previously provided reliable ratings for creativity tasks such as the AUT (Hass et al., 2018; Hass & Beaty, 2018). During instruction, they were told that the responses they would rate came from “MTurkers who were asked to generate the most creative words they could, but linked to a given noun. Creative words are clever or surprising words that very few other people come up with.” Raters were given practice items with feedback to maximize reliability across raters. For example, if they rated the example item *glass-jaw* as low in originality, they received feedback saying “incorrect.” Once they completed the instruction phase, they were given a spreadsheet with a list of responses to rate. Raters coded the responses for originality using a 1 (*low originality*) to 5 (*extremely original*) scale. A composite average of the three raters’ scores was computed for analysis.

## Results

Table 6 presents zero-order correlations and descriptive statistics for creativity ratings and semantic distance models. A total of 1,856 noun responses were included in the analysis. The response set was cleaned for spelling errors and inappropriate responding (i.e., proper nouns). Interrater reliability of responses assessed by the three raters showed excellent agreement (Cronbach’s alpha = .88).

**Table 6:** Study 5 descriptive statistics and correlations of human ratings and multiplicative semantic distance models

	1	2	3	4	5	6	7	8	9	10	M	SD
1. r1_originality	-										1.52	0.42
2. r2_originality	0.89	-									1.92	0.58
3. r3_originality	0.74	0.61	-								2.89	0.55
4. mean_originality	0.95	0.92	0.87	-							2.11	0.47
5. cbu	0.75	0.69	0.80	0.82	-						0.74	0.04
6. cbs	0.72	0.68	0.80	0.81	0.92	-					0.82	0.04
7. cbw	0.68	0.67	0.72	0.76	0.87	0.93	-				0.82	0.04
8. tasa	0.69	0.66	0.77	0.78	0.87	0.84	0.83	-			0.90	0.03
9. glov	0.77	0.68	0.67	0.77	0.89	0.85	0.82	0.79	-		0.76	0.05
10. semdis_factor	0.78	0.73	0.80	0.85	0.97	0.96	0.94	0.89	0.93	-	0.00	0.34

*Note*. *N* = 58; all correlations are significant at *p* < .001. glov = GloVe semantic distance; tasa = TASA semantic distance; cbw = continuous bag of words, Wiki concatenation, semantic distance; cbs = continuous bag of words, ukwac and subtitle corpus, semantic distance; cbu = continuous bag of words, subtitle corpus, semantic distance; r1_novelty-r3_novelty = rater 1-3, novelty rating

### Correlations between human ratings and semantic distance

We began by computing Pearson correlations between the mean originality ratings and the five semantic distance models. All five semantic models correlated highly and positively with originality ratings: cbowukwac (*r* = .82), GloVe (*r* = .77), cbowsubs (*r* = .81), cbowBNC (*r* = .76), and TASA (*r* = .78). Notably, raters often agreed more strongly with individual semantic models than they did with other raters (see Table 6); for example, rater 3 showed the highest correlation with cbowukwac (*r* = .80) and the lowest correlation with GloVe (*r* = .67), whereas rater 1 showed the highest correlation with GloVe (*r* = .77) and lowest correlation with cbowBNC (*r* = .68). This suggests that the five semantic models capture non-redundant variance in human originality ratings. Consistent with this observation, we found a large correlation between mean originality and latent semantic distance (*r* = .85), indicating a strong correspondence between the common variance associated with human originality ratings and semantic distance.

## General discussion

Creativity research has long relied on the subjective judgements of human raters to evaluate the novelty and utility of ideas and products. Although such manual scoring approaches have proved useful for the field, they face two key limitations (labor cost and subjectivity) which threaten reliability and can act as a barrier for researchers without the resources to code thousands of responses. We sought to address these limitations of subjective scoring by capitalizing on recent progress in the automated assessment of creativity via semantic distance. In five studies, we demonstrate that a latent semantic distance variable—reflecting the common variance of five multiplicative compositional models—can reliably predict human judgements on a widely used task of divergent thinking (i.e., the AUT) and on two newly developed word association tasks. Evidence for the convergent validity of semantic distance was found across three studies that included other creativity measures: AUT semantic distance correlated positively with some established measures of creativity, including cognition, personality, and behavior. Together, these findings indicate that semantic distance provides a reliable and valid alternate to human creativity ratings.

Study 1 established evidence for the utility of semantic distance in predicting human creativity on the AUT. Approximately 80% of the variance in human ratings could be explained by a higher-order latent variable comprised of two AUT items and five multiplicative compositional models. These findings are consistent with the recent work of Dumas et al. (2020), who found that human creativity ratings on the AUT correlated strongly with semantic distance, particularly GloVe. Notably, our study used multiplicative models, whereas Dumas and colleagues used additive models, and we found substantially better prediction of human ratings compared to additive models—a finding that replicated in Study 2 and is consistent with recent work comparing additive and multiplicative models in the context of predicting human similarity judgments (Mitchell & Lapata, 2010). In addition, Forthmann et al. (2018) showed that additive compositional models penalize, that is, reduce the semantic distance of longer creative responses, when in fact elaboration should often increase creativity. While removing stop words mitigates this penalty (Forthmann et al., 2018), for the first time, we showed that a multiplicative compositional model reversed the correlation between elaboration and semantic distance: a multiplicative model showed positive correlations between semantic distance and elaboration and an additive model showed negative correlations. More work will be needed to more systematically investigate the role elaboration bias may still play in semantic distance^2^, but we show that employing a multiplicative model substantially improved the correspondence between human creativity ratings and semantic distance. Study 3 replicated the semantic distance-human rating effect with a new and commonly used AUT object (i.e., brick); here, the correlation between semantic and human scores was smaller than Study 1 and 2—due to the use of a single task in Study 3, which highlights the benefits of using multiple trials/tasks to assess creative potential (Barbot, 2018).

It is important to mention that, although semantic distance explained a sizeable proportion of variance in human creativity ratings on the AUT, a non-negligible proportion of variance was left unexplained, potentially due to human raters weighting other factors than novelty (e.g., cleverness, usefulness) when rating alternative uses for objects. In our studies, raters were instructed to prioritize novelty and remoteness (see Supplemental Materials), likely boosting observed correlations with semantic distance. But because the AUT requires people to produce a workable use for an object, raters should also consider the usefulness/appropriateness. Indeed, the semantic distance approach with the AUT can be “hacked” if participants simply respond with random or task-unrelated words, which would yield highly semantically distant but meaningless responses. We therefore encourage users to carefully screen their response files during the preprocessing stage to ensure data quality (a procedure that is notably not necessary for human ratings). In addition, Heinen and Johnson (2018) showed that by simply emphasizing the goal of the task is to “be creative,” participants will implicitly ensure their response are both and appropriate.

Another goal of this research was to assess the convergent validity of semantic distance in the context of the AUT. Previous studies using the verb generation task have found that semantic distance correlates with a battery of established creativity measures (human creativity ratings on the AUT, creative achievement, creative writing) and other cognitive/personality variables linked to creativity (openness, fluid intelligence; Prabhakaran et al., 2014). Our three studies with the AUT provide a partial replication and extension of this work across a diverse range of cognitive and self-report measures. Regarding cognition, Study 1 and Study 3 found that AUT semantic distance correlated positively and moderately with human ratings of creative metaphor quality. Regarding self-report, the findings were more variable across studies. Semantic distance positively predicted openness to experience in two out of three samples, whereas semantic distance predicted creative behavior in one out of two samples. These mixed findings could be explained in part by the inherent limitations of the AUT: although AUT semantic distance did not significantly relate to creative achievement in Study 2, neither did AUT human rating. Moreover, the scale used to assess creative achievement in Study 2 (i.e., the CAQ) typically yields a highly skewed distribution in younger, college samples (Silvia, Wigert, Reiter-Palmon, & Kaufman, 2012), who’ve had little time to produce publicly-recognizable creative products. Nevertheless, we found additional evidence of validity when the focus was more local to the AUT: a semantic distance factor correlated positively with participants’ self-ratings of creativity (Study 2) and it correlated positively with increasing time on task (i.e., the serial order effect; Study 3), indicating that this automated metric captures information important to the task. We also found mixed evidence for the association between semantic distance and fluid intelligence. A notable difference between Study 1 and Study 3, however, concerns the duration of the AUT trials (3 min vs. 10 min, respectively). One possibility is that, consistent with the serial order effect, the greater number of more distant responses in Study 3 provided more variance in performance, increasing the power to detect an effect. Future work should further examine the link between semantic distance and intelligence, employing experimental manipulations of task parameters (e.g., trial duration) to determine the extent to which the ability to generate semantically-distant ideas relates to fluid intelligence.

Study 4 and Study 5 extended our application of semantic distance to two word association tasks: the verb generation task (Study 4; Prabhakaran et al., 2014) and the C-BAND (Study 5; Johnson et al., 2019). Study 4 found that, across two samples, the five semantic models correlated positively (but variably) with human ratings of novelty, appropriateness, and creativity. The highest correlations were found for novelty, with correlations between the semantic distance and human novelty ratings approaching unity in sample 2. Interestingly, despite relatively high inter-rater agreement, the human raters often agreed more with individual semantic models than with other human raters. In a similar vein, specific raters tended to correlate more with some semantic models than others, suggesting that the five semantic models capture nonredundant variance in human judgements and lending support for a latent variable approach. Moreover, Study 5 found a large correlation between a latent semantic distance factor and a latent variable comprised of human originality ratings (*r* = .85), approaching the near-perfect correlation between semantic distance and novelty ratings found in Study 4. This finding illustrates the importance of instruction at both the front-end (participant) and back-end (rater): when novelty is emphasized over appropriateness, the correlation between human ratings and semantic distance will likely increase. But with a greater emphasis on appropriateness, the correlation is likely to be attenuated, consistent with the increasing pattern of correlation reported in Study 4 for appropriateness, creativity, and novelty, respectively (cf., Heinen & Johnson, 2018).

### Summary, limitations, and future directions

The present study is the first, to our knowledge, to leverage latent variable modeling to combine multiple semantic distance models in the context of creativity assessment (cf. Beketayev & Runco, 2016). Recent work has provided evidence for the utility of individual semantic models in predicting human ratings of creativity (Dumas et al., 2020; Johnson et al., 2019; Prabhakaran et al., 2014), with a majority of work focusing on the TASA model of LSA (Kenett, 2019). A strength of this approach is that it can address some previous limitations of semantic distance applications to creativity research, such as biases introduced by corpus choice and algorithms used to compute semantic distance. Although the current approach is not immune to such limitations, the inclusion of several of the top-performing models currently available—which include corpora from diverse sources of naturalistic language (e.g., subtitles)—partially mitigates this source of bias. It is important to note, however, that some of the text corpora used in the current study overlapped (i.e., the three CBOW spaces shared some of the same texts), which may have influenced the current findings; indeed, it is possible that one could achieve comparable validity with a more simple composition of semantic spaces, such as the recent validation study by Dumas et al. (2020), which showed similarly high correlations between human ratings and semantic distance with single semantic models.

Although we generally recommend that future users adopt the latent variable approach, there may be some cases where a specific semantic model (or an average of the five models’ semantic distance scores) would be best. Factor models require large amounts of data for model convergence and reliability (*n* > 100). These issues and others can also lead to less than adequate fit of the data to the specified structural models, as was the case with some models presented in the present study, which occasionally yielded fit statistics above recommended cutoffs. Consequently, if data sets are small, then we would recommend using a single semantic model or averaging the semantic distances scores across the five semantic models. We therefore included a feature on the online platform (*SemDis*) that gives users the option to extract and download a latent factor score comprised of the five semantic models (see Supplemental Tutorial) alongside semantic distance scores from the five individual models and an average semantic distance score across the five models.

The present work contributes to the growing study of creativity in the context of semantic networks (Christensen & Kenett, 2019; Kenett & Faust, 2019; Zemla, Cao, Mueller, & Austerweil, 2020). Kenett and colleagues have published several recent papers empirically validating the longstanding associative theory of creativity (Mednick, 1962), which posits that creative thinking involves making connections between remote concepts in semantic memory. Several studies have found that individual creative thinking is characterized by a more flexible network structure, marked by short path lengths and high connectivity between nodes, coupled with low modularity of the network structure; these networks can be modeled by applying network science tools to free association data (Kenett et al., 2014) and human relatedness judgements (Kenett, Levi, Anaki, & Faust, 2017), which can in turn be related to measures of creative thinking (Christensen et al., 2018; Kenett, 2019; Kenett et al., 2014; Kenett & Faust, 2019). One mechanism thought to facilitate conceptual combination is spreading activation—activation of one concept in semantic memory spreads to other connected concepts and quickly decays over time. De Deyne and colleagues proposed a spreading activation metric derived from word association data (De Deyne, Navarro, Perfors, & Storms, 2016), which they showed was capable of capturing weak but reliable similarity judgments. Future research could explore whether this approach can complement other semantic measures to quantify individual differences in creative thinking along the lines of the current study and the network-based methods of Kenett et al.

It is important to consider the limitations of semantic distance for creativity assessment. Although the semantic distance approach provides a useful tool for creativity research, it may not be necessarily more reliable or valid than subjective ratings. Along these lines, we found that human ratings tended to show numerically higher validity with respect to correlations with other creativity measures. Importantly, however, we also found that semantic distance reliably correlates with these same measures, suggesting that this automated approach provides a reliable and valid alternate to human ratings. Another notable feature of semantic distance is its relative correspondence to human novelty vs. creativity ratings. Indeed, our data suggest that semantic distance is slightly more sensitive to novelty than creativity, consistent with the similarity-based algorithms used to compute these values. A semantically-distant response is thus likely to be perceived by humans as novel because both humans and semantic models are sensitive to conceptual remoteness. But the creativity criterion has the added burden of usefulness, i.e., whether the response is fitting, witty, or clever, which is not currently captured by semantic distance. In the end, semantic distance is a novelty metric, and not a direct line to creativity—only a proxy with demonstrable validity. At the same time, we would argue that undergraduate students, who often rate responses to creativity tasks, are also not a direct line to creativity. Indeed, past work has highlighted issues with their data as well (e.g., fatigue, bias, disagreement, etc.). Moreover, creativity researchers do not all agree on what constitutes a creative idea, so semantic distance and human raters may both be imperfect, just in different ways. Ultimately, given the burdens of subjective human ratings, if automated assessments even come close to the levels of validity of human ratings, we see this is a substantial step forward.

We encourage future research to continue to explore automated approaches to creativity assessment. Indeed, we encourage active debate on the limits on such automated tools. Can computational tools perfectly capture human creativity? Are there some cases where human judgements are preferred over computational metrics? The current study indicates that computational linguistic measures of semantic distance can explain considerable variance in human creativity and novelty ratings, but our findings are limited to verbal creativity and word association tasks. The extent to which automated methods can capture creativity in the context of non-verbal tasks (e.g., drawing) remains unknown. To this end, future work could leverage machine learning methods to uncover the features of visuospatial creative products that predict human creativity judgements. Moreover, semantic distance is best suited to capture novelty, but creativity is thought to require both novelty and usefulness (Diedrich et al., 2015). The semantic distance approach could thus be supplemented in the future by adding an algorithm that weighs novelty and usefulness similar to how people do when making aesthetic judgements, which may bring us closer to achieving a fully automated assessment of human creativity.

## Electronic supplementary material

ESM 1 (DOCX 35 kb)

## Footnotes

1 The file size limitations and current computational processing speed for SemDis can be found in the SemDis Tutorial tab (Troubleshooting and File Size Limits) of the webapp at semdis.wlu.psu.edu.

2 We thank a reviewer for highlighting this point and for performing preliminary simulations demonstrating elaboration bias still exists when using multiplicative models.
