# Supplementary material for: Automating creativity assessment with SemDis: An open platform for computing semantic distance
Source: Behav Res Methods. 2020 Aug 31;53(2):757–80. doi: 10.3758/s13428-020-01453-w (PMC8062332; doi:10.3758/s13428-020-01453-w)
Supplement: Supplementary file 1 — (DOCX 35 kb) [file 13428_2020_1453_MOESM1_ESM.docx]

**Supplemental Materials**

**Study 1-3: AUT, Participant Instructions**

CREATIVE USES TASK

For this task, you'll be asked to come up with as many original and creative uses for a BOX as you can. The goal is to come up with *creative ideas*, which are ideas that strike people as clever, unusual, interesting, uncommon, humorous, innovative, or different.

Your ideas don't have to be practical or realistic; they can be silly or strange, even, so long as they are CREATIVE uses rather than ordinary uses.

You can enter as many ideas as you like; just press ENTER after each one.

You can type in as many ideas as you like until then, but *creative quality is more important than quantity*. It's better to have a few really good ideas than a lot of uncreative ones.

**Study 1-3: AUT, Rater Instructions**

Creativity can be viewed as having three facets. Creative responses will generally be high on all three, although being low on one of them does not disqualify a response from getting a high rating. We will use a 1 (*not at all creative*) to 5 (*highly creative*) scale.

***1. Uncommon***

Creative ideas are uncommon: they will occur infrequently in our sample. Any response that is given by a lot of people is common, by definition. Unique responses will tend to be creative responses, although a response given only once needn’t be judged as creative. For example, a random or inappropriate response would be uncommon but not creative.

***2. Remote***

Creative ideas are remotely linked to everyday objects and ideas. For example, creative uses for a brick are “far from” common, everyday, normal uses for a brick, and creative instances of things that are round are “far from” common round objects. Responses that stray from obvious ideas will tend to be creative, whereas responses close to obvious ideas will tend to be uncreative.

***3. Clever***

Creative ideas are often clever: they strike people as insightful, ironic, humorous, fitting, or smart. Responses that are clever will tend to be creative responses. Keep in mind that cleverness can compensate for the other facets. For example, a common use cleverly expressed could receive a high score.

***Extra Information***

- Quickly read all the responses first to get a sense of commonness and uniqueness trends.
- Give low scores to actual intended uses for the objects (e.g., making a fireplace with bricks).
- Use the whole scale. Save the 1s for the really obvious, terrible, and confused responses.
- Overlook spelling mistakes—people are usually typing quickly.
- Feel free to revise your ratings after judging everything. After rating everything, for example, it’s a good idea to sort “descending” by your ratings, and see if you want to change any of your higher scores to be even higher. In hindsight, many will look better, and some will look worse.

**Study 5: C-BAND, MTurk Rater Instructions**

The data you will rate comes from other MTurkers who were asked to generate the most **CREATIVE words** they could, but linked to a given noun. Creative words are clever or surprising words that very few other people could come up with.

For example, an MTurker receives the noun, **glass**, and generates the response, **jaw**.

You would rate this noun-response word pair, that is, glass-jaw on its **ORIGINALITY**. So, evaluate how original or clever you believe the response, jaw, is. To be extremely original (that is, a 5) on the 1-5 scale, means you believe the response is highly original. So you would believe very few other people would come up with it.

Please **do not** review the responses first, just start rating at the top of the column and work your way down. After reading these instructions and doing a couple practice ratings, you will receive a link to a spreadsheet with a **noun**column and a **response**column and a "**Your ORIGINALITY Rating (1-5 scale)**" blank column. The **noun**is what we gave the MTurker. They were asked to generate a creative word in response to this given noun in the **response**column.

You will rate the originality of each noun-response pair on a 1-5 scale, where a**5 is the MOST original. Just type the number corresponding to your rating in the cell of the spreadsheet.**

Here is the scale you should use:

1 = low originality

2 = slightly original

3 = moderately original

4 = very original

5 = extremely original
